# The interrelationship between childhood emotional abuse and aggressive behavior in the Chinese adolescent population: a network perspective

**DOI:** 10.1186/s13034-025-00931-3

**Published:** 2025-06-14

**Authors:** Yuhao Wang, Yuxuan Liu, Susu Tian, Xinyi Hu, Jiayi Tian, Yingxue Wang, Yihan Wang, Chunxia Miao, Wei Wang

**Affiliations:** 1 School of Public Health, Xuzhou Medical University, 209 Tong Shan Road, Xuzhou, 221004 Jiangsu China; 2 Department of Public Health, The Affiliated Xuzhou Oriental Hospital of Xuzhou Medical University, Jiangsu Xuzhou, China; 3 Research Center for Psychological Crisis Prevention and Intervention of College Students in Jiangsu Province, Xuzhou Medical University, Xuzhou, China; 4 Jiangsu Engineering Research Center of Biological Data Mining and Healthcare Transformation, Xuzhou Medical University, Jiangsu 221004 Xuzhou, China; 5 School of Management, Xuzhou Medical University, 209 Tong Shan Road, Xuzhou, Jiangsu China

**Keywords:** Rural adolescents, Emotional abuse, Aggressive behavior, Network analysis

## Abstract

**Background:**

Numerous studies have found a strong correlation between emotional abuse in childhood and aggressive behavior in adolescents, especially among rural youth. However, the complex relationship between the different sub-dimensions of aggression and emotional abuse is unclear. This study aimed to explore the association between emotional abuse and different dimensions of aggressive behavior in rural adolescents using network analysis.

**Methods:**

The participants in this study came from multiple middle schools and a total of 1797 adolescents were included in the study. Questionnaires were self-reported using the CTQ-SF (Childhood Trauma Questionnaire-Short Form) scale and Buss and Warren aggression questionnaire. A network analysis was performed.

**Results:**

The network analysis revealed that hostility and anger were the most central dimensions of aggression, with " Sometimes I feel like people are laughing at me behind my back " and " I have a hard time controlling my temper " being the most influential nodes. Emotional abuse symptoms, particularly “Someone in my family has said insulting or sad things to me,” served as critical bridge symptoms, linking emotional abuse to aggressive behavior. Gender differences were significant, with males exhibiting stronger associations between emotional abuse and physical aggression, while females showed stronger links between emotional abuse and internalizing symptoms like hostility and anger.

**Conclusion:**

This study highlights the central role of hostility and anger in the relationship between emotional abuse and aggressive behavior among rural adolescents. Poor family relationships, particularly insulting or demeaning remarks from family members, were identified as key bridge symptoms that exacerbate aggressive tendencies. Gender-specific patterns suggest that interventions should be tailored to address physical aggression in males and internalizing symptoms in females.

**Supplementary Information:**

The online version contains supplementary material available at 10.1186/s13034-025-00931-3.

## Introduction

Childhood trauma has been widely studied for its adverse impacts(Bahk et al. 2017; Rhee et al. 2019; Wan et al. 2019), including increased risks of traumatic stress disorder, depression, cardiovascular diseases, obesity, aggression, and reduced social adaptability in adolescents, with effects persisting into adulthood(Hoeboer et al. 2021; Humphreys et al. 2020; Rothenberg et al. 2019; Scheuer et al. 2018; Wan et al. 2019; Zhang et al. 2018). These consequences exhibit gender differences(O’Shields and Gibbs 2021; Warren et al. 2019). For example, males often externalize trauma through aggression, while females tend to internalize distress through depression, anxiety, or physical manifestations like Premenstrual Syndrome(Bjorkqvist 2018; Hofmeister and Bodden 2016; Holder and Blaustein 2014; McCarron et al. 2021). Rural adolescents, particularly in China, face heightened vulnerability due to systemic inequalities exacerbated by urbanization and the hukou system, which restricts rural residents’ access to education, healthcare, and social services(Liang et al. 2019; Wen et al. 2021; Zhang et al. 2020). Additional challenges include high rates of parental migration (e.g., left-behind children), limited mental health resources, and cultural norms prioritizing academic achievement over emotional well-being(Chen et al. 2019; Wen et al. 2021), making this group disproportionately exposed to childhood trauma and its long-term repercussions.

Particularly relevant to aggression, the relationship between childhood trauma and adolescent aggressive behavior has been extensively documented(Augsburger et al. 2019; Debowska et al. 2018; Kang et al. 2021; King et al. 2019; O’Shields et al. 2022). However, most studies focus on the overall trauma score rather than one of its five dimensions -emotional abuse, as defined by the Childhood Trauma Questionnaire(Aloba et al. 2020; Bernstein et al. 1994). Several global meta-analytic types of research display that the prevalence of emotional abuse ranks first among the five dimensions, reaching as high as 36%(Wang et al. 2020). Relevant studies show that compared with other maltreatment subtypes, emotional abuse is more closely related to psychological internalization symptoms in adolescents(Cohen and Thakur 2021), and is more likely to lead to the occurrence of aggressive behavior(Ran et al. 2023), yet its specific role in aggressive behavior remains understudied despite its high incidence(Gu et al. 2020; Maxwell 2008).

Among these problems caused by emotional abuse, aggressive behavior is an area of increasing research interest. Aggressive behavior refers to any action with the immediate intent to harm or hurt another individual, including physical and psychological damage(Bushman and Anderson 2001). Globally, aggressive and violent behavior has become a significant public health and social problem in adolescents, with estimates placing the number of deaths, nonfatal injuries, and mental health issues caused by this behavior at 1.4 million per year(Kang et al. 2021). Multiple studies indicated that adolescents with higher levels of aggressive behaviors might be at increased risk for self-injury or suicide(Holt et al. 2015; Lear et al. 2020; Zhang et al. 2018). Rural adolescents with high levels of aggressive behaviors are more likely to drink, break the law, and even commit crimes in the future than urban adolescents who are equally aggressive(Lear et al. 2020; Smokowski et al. 2017). Furthermore, research suggests that rural adolescents are more likely to engage in aggressive and violent behavior than urban adolescents, and aggressive behavior is prevalent among adolescents in rural China(Huang et al. 2017). It is essential to pay more attention to aggressive behavior and its influencing factors among rural adolescents.

Network analysis is a practical approach to investigating complex, dynamic relationships between individual psychiatric and behavioral symptoms(Fisher et al. 2017). Unlike conventional methods that prioritize linear relationships or latent constructs, network analysis models individual psychiatric and behavioral symptoms as dynamic systems, where nodes reflect specific symptoms, and edges between nodes reflect relationships between symptoms, including the activation spread from one sign to another through the network(Borsboom 2017). Nodes can also function as bridge symptoms that transfer symptom activation from one disorder to another. Therefore, network analysis is particularly effective in elucidating connection patterns between individual psychiatric symptoms, behaviors, and disorders, enabling precise identification of how specific emotional abuse subtypes drive aggressive behaviors through symptom activation pathways(Borsboom 2017; Rouquette et al. 2018). No team has yet to conduct a network analysis of childhood trauma and aggressive behavior among rural middle school students. This study aims to fill this gap.

Therefore, this study aimed to characterize the network structure of adolescent emotional abuse and aggressive behavior among rural adolescents, explore the nodes that play a prominent role in the network, understand the differences in aggressive behavior between the genders, and identify and propose target interventions for the most acute symptoms that affect emotional trauma and aggressive behavior. Ultimately, the study has the potential to improve the mental health of rural adolescents and provide a basis for improving school counseling services.

## Methods and materials

### Participants

In this study, four rural middle schools in Xuzhou City, Jiangsu Province, were randomly selected using multi-stage whole-group sampling. All students in the selected classes were included in the study based on stratification by school grade and through the principle of random class selection. A total of 2004 questionnaires were distributed, and 1920 questionnaires were returned, yielding a recovery efficiency of 95.81%. Based on the purpose and need of the study, after eliminating the questionnaires containing missing values of relevant variables, 1797 questionnaires were finally collated and applied to this study.

### Measures

#### Demographic characteristics

Before implementing the survey in this study, the subjects provided informed consent. Gender, age, grade level, whether they were only children, and whether they were left-behind children were collected as basic demographic information.

#### Child emotional abuse

We used the CTQ-SF (Childhood Trauma Questionnaire-Short Form) scale for childhood trauma data collection. The scale is a self-administered questionnaire that retrospectively assesses the experience of childhood maltreatment and is prepared by Bernstein & Fink (1994)(Aloba et al. 2020; Bernstein et al. 1994; Bernstein et al. 2003). There were 25 clinical items and three validity items related to five specific types of childhood trauma: emotional abuse, emotional neglect, physical abuse, physical neglect, and sexual abuse. Each question was measured using a 5-point Likert-style response, indicating the frequency of a specific childhood trauma scenario(Bernstein et al. 1994). The total CTQ score is derived from the total score of the five subscales, with higher scores meaning higher levels of childhood trauma. For this study, we used data from the child emotional abuse subscale. In the Chinese population, the instrument was reported to have high reliability and validity(He et al. 2019).The Cronbach’s α of the scale was 0.71, suggesting good reliability.

#### Adolescent aggressive behavior

We used the Buss and Warren aggression questionnaire for adolescents’ aggressive behaviors data collection(Bernstein and Gesn 1997; Buss and Perry 1992; Maxwell 2008). The questionnaire has five dimensions: physical aggression, verbal aggression, anger, hostility, and indirect aggression, which measure adolescents’ tendencies to behave aggressively. The questionnaire has 34 entries and uses a 5-point scale, with higher scores indicating that the test subject also has a higher propensity to be aggressive. Cronbach’s α was 0.82 in this study, suggesting good reliability.

### Analytical strategies

#### Network estimation

All analyses and network model were computed using R-studio (version 4.3.1). Paired Spearman correlation analysis estimated the symptom network illustrating the relationship between symptoms. In the network analysis method, each symptom is treated as a node, and the pairwise correlation between these nodes are treated as edges(Borsboom and Cramer 2013). We estimated the Graphical Gaussian Model (GGM), with the graphic least absolute shrinkage and selection operator (LASSO) and Extended Bayesian Information Criterion (EBIC) model using R package “graph”(Epskamp et al. 2018).

Strength was used as a centrality indicator to investigate the significance of individual symptoms in the network. Strength is the sum weight of all direct relationships between a particular sign and other symptoms. Centrality measures are reported as standardized values (z-scores). The bridge function in R-package “networktools” was also used to investigate network bridge symptoms essential in linking two or more mental diseases(Cramer et al. 2010). Use bridge strength to assess the centrality of bridge symptoms. Based on previous investigations, bridge symptoms were chosen using an 80th-percentile bridge strength limit(Jones et al. 2021). In order to investigate the direct link between the most central symptom in the network and the other symptoms, the “flow” function in R package “qgraph” was used(Epskamp et al. 2012).

#### Estimation of network accuracy and stability

The node and bridge strength stability were assessed using the R-package “bootnet” and a case-dropping bootstrap procedure. This procedure removed many cases from the dataset and recalculated the centrality indices. Suppose most samples can be removed from the dataset without significantly affecting the node’s centrality index. In that case, the network is said to be stable, and the stability is quantified by the Correlation Stability Coefficient (CS-C). CS-C refers to the maximum number of cases that can be excluded from the sample. The centrality indices from the subsamples are correlated with the index from the original selection at a value of *r* = 0.7 (Epskamp et al. 2018). In general, CS-C should be greater than 0.25, preferably greater than 0.5. The confidence interval (CI) for the correctness of an edge weight was computed using a non-parametric bootstrap procedure. The observations are randomly resampled to generate new data sets from which a 95% CI can be calculated, with a narrower CI indicating a more trustworthy network. To analyze variations in network characteristics, we performed a bootstrap differential test with 1,000 permutations and ran the results.

#### Network comparisons

Study used Network Comparison Tests (NCT) to explore whether there were differences in network structure between gender. This is a permutation test to compare the differences between two networks. NCT is performed on a sub-sample of the residency definition using 1000 permutations, a technique that compares the absolute sum of all edge weights between networks to determine the overall strength of the network. The distribution of edge weights in each network is then compared to determine the network’s structure. The analysis was done with the “NetworkComparisonTest” R package(van Borkulo et al. 2022).

## RESULT

### Descriptive statistics

Table 1 presents the demographic characteristics of the participants and specific scores for emotional abuse and aggressive behavior. Of the 1797 rural middle school students included in this network analysis, the mean age was 16.3 years (SD = 1.628), and 786 (43.74%) were males. The junior high school has 773 students, and the senior high school has 1024 students. Of all the students included in the survey, only 181(10.1%) were only children. There were 493 students whose fathers worked as migrant workers and 31 students whose mothers worked as migrant workers. 135(7.5%) students were left-behind children, and this percentage is smaller compared to rural areas in other parts of China(Wen et al. 2021).

**Table 1:** Demographic characteristics of the participants and specific scores for emotional abuse and aggressive behavior

Variables	Total		Male		Female	
	Mean/*N*	SD/%	Mean/*N*	SD/%	Mean/*N*	SD/%
Participants	1797	-	786	43.74	1011	56.36
**Age**	16.30	1.628	16.16	1.709	16.40	1.555
Grade						
Junior high school students	773	43.1	390	49.6	383	37.9
Senior high school students	1024	56.9	396	50.4	628	62.1
Only child						
Yes	181	10.1	108	13.7	73	7.2
No	1616	89.9	678	86.3	938	92.8
Parents go out to work						
Only father	493	27.4	217	27.6	276	27.3
Only mother	31	1.7	11	1.4	20	2
All	135	7.5	73	9.3	62	6.1
No	1138	63.3	485	61.7	653	64.6
Left-behind child						
Yes	135	7.5	73	9.3	62	6.1
No	1662	92.5	713	90.7	949	93.9
Questionnaire scores						
Emotional abuse	6.30	2.137	6.21	2.227	6.36	2.063
Aggressive behaviors	70.34	19.292	71.09	21.196	69.75	17.66
Physical Aggression	12.85	5.061	14.16	5.922	11.83	3.988
Verbal Aggression	12.06	3.631	12.37	3.927	11.83	3.367
Indirect Aggression	11.69	4.097	11.65	4.483	11.73	3.771
Anger	15.86	5.357	15.43	5.269	16.2	5.403
Hostility	17.87	6.222	17.49	6.444	18.17	6.031

(*N* = 1797)

### Network estimation and centrality measure analysis

**Fig. 1:**
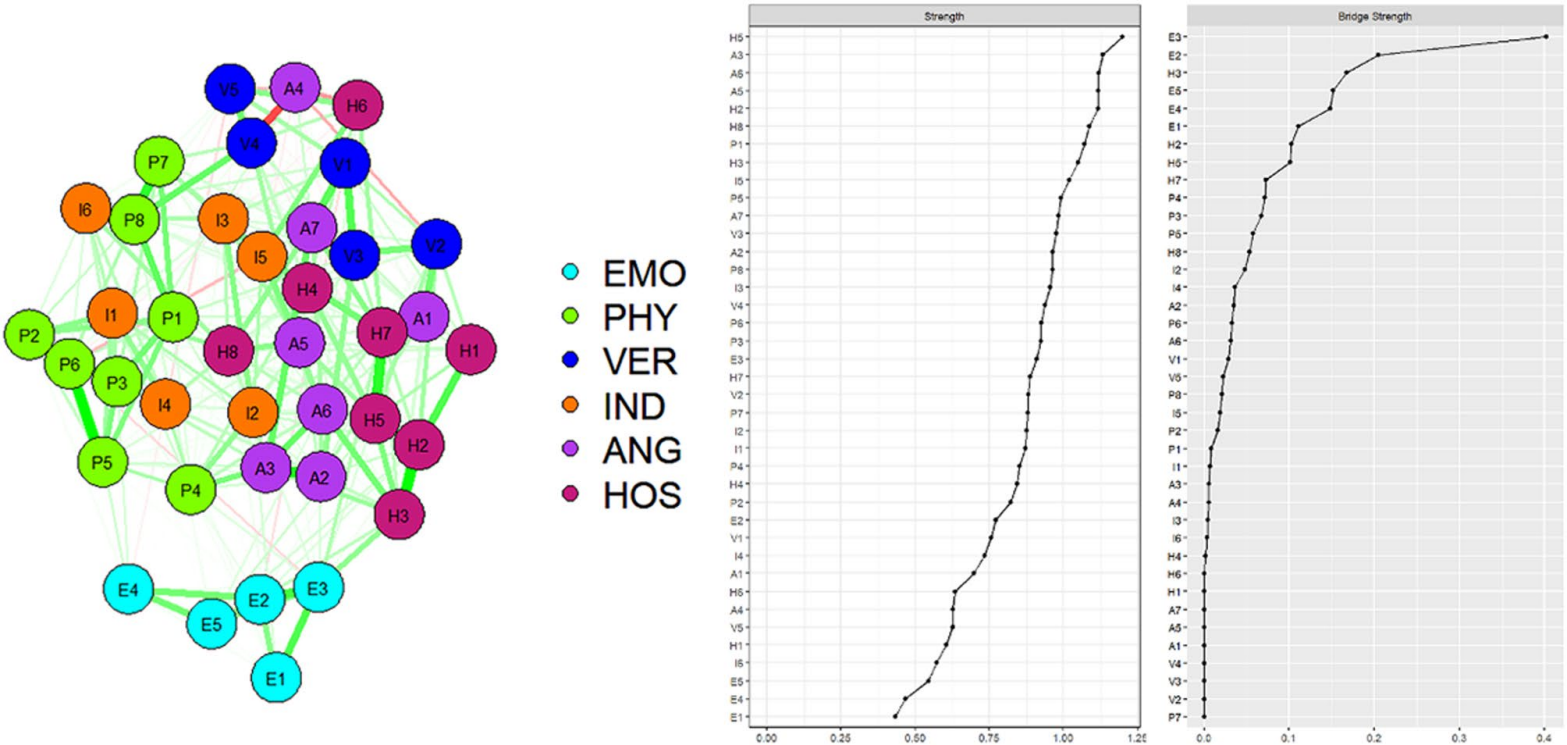
The network structure of emotional abuse-aggressive behaviors in rural adolescence. EMO: Emotional abuse; PHY: Physical Aggression; VER: Verbal Aggression; IND: Indirect Aggression; ANG: Anger HOS: Hostility; The symptoms represented by each node abbreviation refer to Supplementary Table 1

Figure 1 depicts the emotional abuse and aggressive behaviors symptom network and the centrality measures of all the symptoms. The majority of identified associations were positive, and 333 edges were estimated to be non-zero. The composite network structure show that the edge H2(I do not know why I feel so miserable sometimes) and H3(Sometimes, I think life is unfair to me) have the strongest correlations, followed by the edge A2(Sometimes I get angry for no reason) and A3(I have a hard time controlling my temper). Overall, emotional abuse symptoms were positively associated with aggressive behaviors. A weighted adjacency matrix was used to examine the numerical interactions between these symptoms and the results are shown in **Supplementary file 1**. Node H5(Sometimes I feel like people are laughing at me behind my back) is the most influential in the network, followed by A3(I have a hard time controlling my temper), A6(Many times I feel like a bomb going off). In contrast, the influence of E4 (Someone in my family hates me), and E1 (Some people in the family are called “lazy pig” or “ugly” and other unpleasant terms) is minimal.

**Fig. 2:**
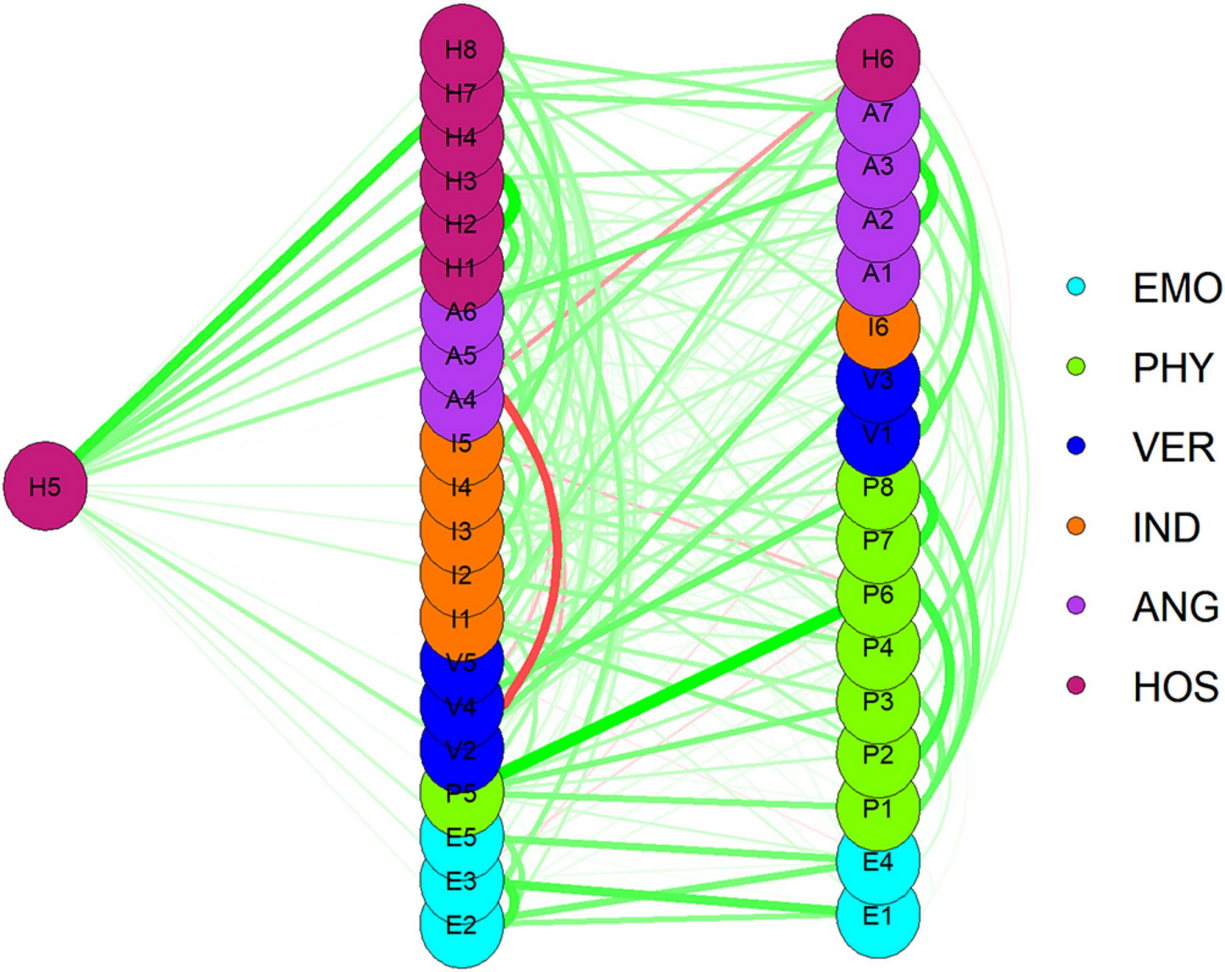
Flow network of “Sometimes I feel like people are laughing at me behind my back”

Based on previous research findings, the bridge strength of a node can effectively reveal the impact of this node on surrounding nodes. (Garabiles et al. 2019; Jones et al. 2021). As shown in Fig. 1, in terms of bridge symptoms, E3 (Someone in my family has said insulting or sad things to me), E2(My parents wish they had never had me), and H3(I do not know why I feel so miserable sometimes) were the most prominent bridging symptoms in this study. The center node of the network is H5(Sometimes I feel like people are laughing at me behind my back), and the flow network highlights connectivity within the network: the edge between nodes H5 and H7(I know there are so-called “friends” talking about me behind my back) reflected the strongest connection, followed by edge between H5 and H3(I don’t know why I feel so miserable sometimes) (Fig. 2).

### Network accuracy and stability

**Fig. 3:**
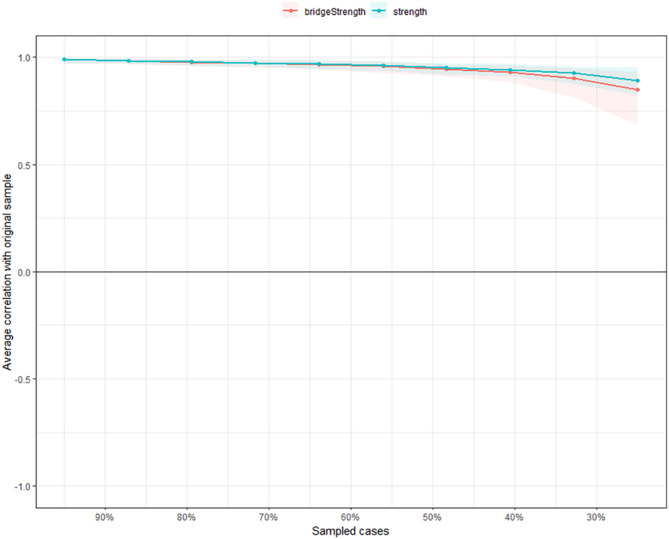
The Stability of centrality indices by case-dropping bootstrap. Note: The stability of strength and bridge strength using case-dropping bootstrap. The x-axis indicates the percentage of cases of the original sample included at each step. The y-axis indicates the average correlations between the centrality indices from the original network and the centrality indices from the networks that were re-estimated after excluding increasing percentages of cases. Each line indicates the correlations of strength and bridge strength, while areas indicate 95% CI

As shown in Fig. 3, we analyzed the stability of the network and found it to be very stable. The case-dropping bootstrap procedure resulted in CS-Cs of 0.75 for node strength, indicating that 75% of the samples could be removed without significant changes in the network structure compared to the original structure. The edge weights of the current model, especially those with larger weights, are consistent with the edge weights of the bootstrap sample, indicating that the existing network structure is stable (Supplementary Fig. 1). The bootstrap 95% CIs for the estimated edge weights suggest that the network model is reliable and stable. Supplementary Fig. 2 indicated that bootstrap difference tests showed that most edge-weight comparisons were statistically significant. The Bootstrapped 95% CI of the estimated edge weights indicates that the network model is reliable and stable (Supplementary Fig. 3).

### Network comparisons between male and female

**Fig. 4:**
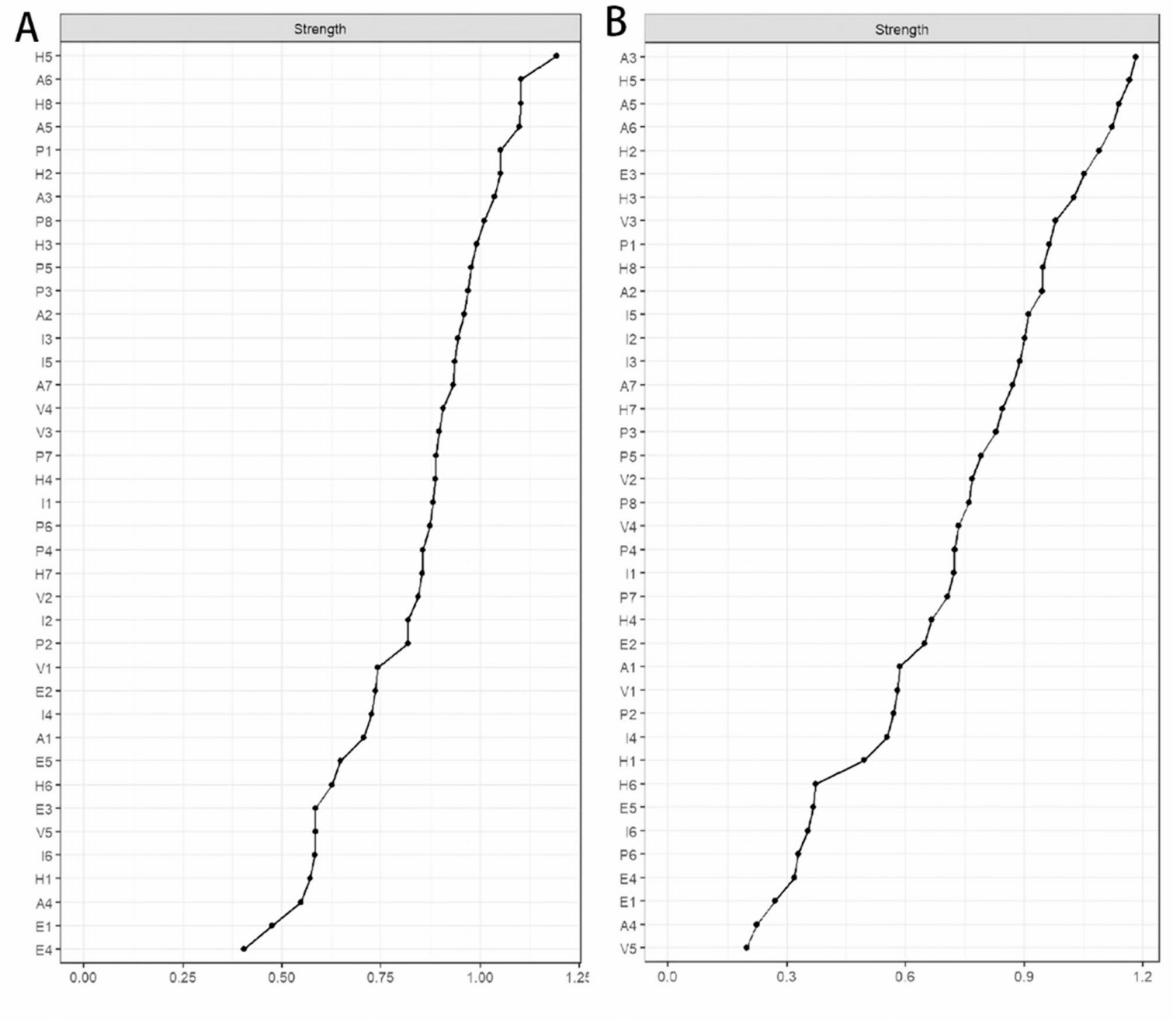
Central symptoms of network structure in different gender populations. Note: Fig. 4.A is the order of node strength in the male network structure, and Fig. 4.B is the female network structure

The result of Network Comparison Tests showed that gender differences significantly affected the emotional abuse and aggressive behaviors symptoms model. There were significant differences in the overall strength of the network(*P* = 0.013) and the distribution of edge weights(*P* = 0.002) in the comparison of network models (Supplementary Fig. 4). Figure 4 shows that the highest node strength in the male network structure was H5(Sometimes I feel like people are laughing at me behind my back). In the female network structure, A3(I have a hard time controlling my temper) ranked first. The weighted adjacency matrix and network models of male and female are shown in **Supplementary file 2 and 3**.

**Fig. 5:**
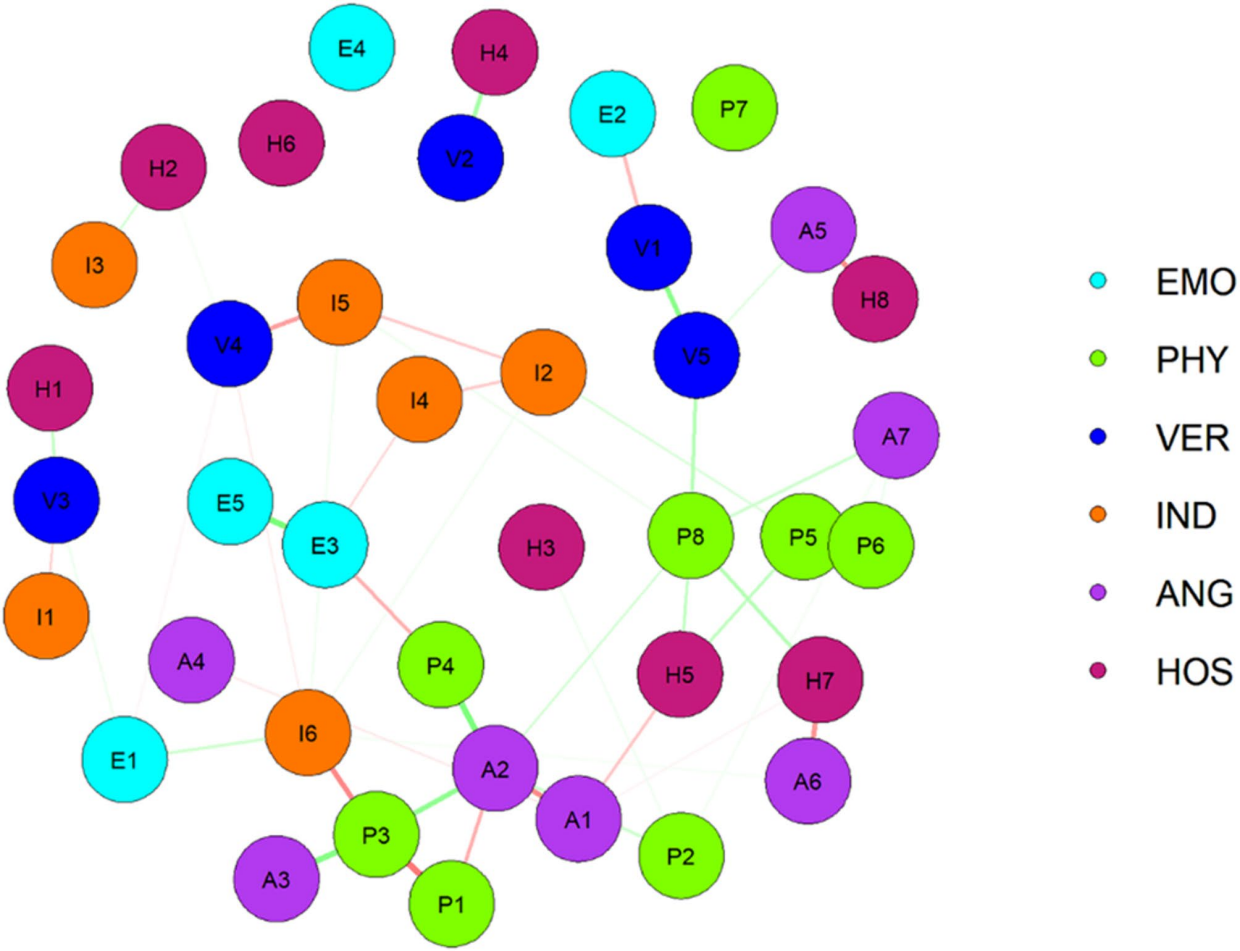
Parts of the network structure that differ between male and female. Note: The green line represents a significantly higher correlation between these two behaviors for males than for females, and the red line represents the opposite

As shown in Fig. 5, the green line between the two nodes indicates that the association between males is significantly more significant than between females. The association between P5(I sometimes hit people uncontrollably) and P6(I fight more than most people) was more significant for males than females. In contrast, the red line segment is the exact opposite. Females are significantly higher than male in the association of P1(If someone provokes me, I might hit him) and P3(If someone pushes me hard, I hit him or her).

## Discussion

To our knowledge, this was the first study that characterized the network structure of child emotional abuse and adolescent aggressive behavior symptoms among rural adolescents. In the current study, we explored the critical nodes in the network of emotional abuse and aggressive behavior in adolescents and also found differences in the network structure by gender.

From the results of the network analysis, we identified anger and hostility as the predominant aggression behavior sub-dimensions. “Sometimes I feel like people are laughing at me behind my back” emerged as the most central symptom in the network, reflecting a cognitive bias likely fueled by low self-esteem and heightened sensitivity(Golec de Zavala et al. 2020). This bias may stem from emotional abuse experiences, which reinforce hypersensitivity to negative social cues. Over time, such perceptions trigger hypervigilance to threat(McLaughlin et al. 2009), fostering hostility as a preemptive defense mechanism. This pathway aligns with the social information processing model(Lansford et al. 2006), where maladaptive cognitive patterns mediate abuse-aggression links. In addition, low self-esteem and sensitivity are substantial psychological problems that plaguing Chinese rural students which are potential causes of depression and aggressive behavior in adolescents(Lai et al. 2021; Yu et al. 2022). Being laughed at behind their backs, whether real or imagined, can be even more traumatizing for rural adolescents who are still mentally immature at puberty and are also in more complex environments, making them feel emotionally abused(Chen et al. 2022). Studies on adolescent aggressive behavior have shown that a teenager with a higher psychological burden is associated with a higher total aggressive behavior(Mazefsky 2022; Vega et al. 2022). Furthermore, symptoms like “I have a hard time controlling my temper” may reflect emotion dysregulation(Furente et al. 2024), a core mechanism linking emotional abuse to aggression. Chronic exposure to hostile family environments impairs adolescents’ ability to modulate negative emotions, resulting in impulsive anger outbursts. This aligns with the emotional flooding hypothesis(Gottman 1993), where unregulated anger overwhelms cognitive control. Additionally, aggressive behavior often stems from the internalization of psychological burden. These subjective perceptions can intensify negative emotions, leading to externalizing behaviors like aggression as a way to cope with or deflect perceived threats.(Luft et al. 2022). Various adolescent internalizing symptoms, including depression, are associated with aggressive behavior.(Liu et al. 2022; Wang et al. 2015). This finding underscores the importance of subjective social perception in the psychopathology of aggression. For rural adolescents, perceived ridicule is not merely a symptom but a catalyst for broader psychological and behavioral dysfunction(Lin et al. 2022).

Emotional abuse and aggressive behavior in adolescents can interact with each other and lead to increased adverse outcomes(Norman et al. 2012; Wan et al. 2019; Zhang et al. 2020). Bridge symptoms can be viewed clinically as transdiagnostic, and targeted interventions for both disorders may be effective(Kaiser et al. 2021). “Someone in my family has said insulting or sad things to me” and “My parents wish they had never had me” were the significant bridge symptoms between emotional abuse and the aggressive behaviors network we discovered in this study. These symptoms reflect direct verbal abuse and emotional neglect within families, acting as conduits for transmitting trauma to behavioral consequences. Specifically, family relationships emerged as a critical contextual factor: poor family environments directly exacerbate aggressive tendencies and hinder the psychological maturity and development of teenagers(Winding and Andersen 2019), while prior research highlights that positive family relationships promote lifelong mental health(Chen and Harris 2019). To prevent adolescents from suffering from the above problems, we should not only provide psychological counseling and assistance to adolescents, but also strengthen communication with parents and establish a harmonious family environment for them(Chen et al. 2017; Narayan et al. 2021). In addition, the findings suggest that addressing this symptom requires a multilevel approach that targets both individual and family-level factors, with interventions tailored to the unique cultural and socioeconomic context of rural China. By focusing on improving family relationships and providing accessible mental health support, this study can disrupt the cycle of trauma and promote healthier developmental outcomes for rural adolescents.

By comparing the aggressive behavior scale scores of different gender groups, we found that the mean scores of males were higher than those of females, consistent with the results of a previous study(Wu et al. 2020). There was an increase in the intensity of several nodes of physical aggression in male adolescents and the core symptoms in females continued to be dominated by hostility and anger. It can be seen that aggressive behavior in males is more likely to translate into physical conflict(Xie et al. 2011). The key node in the female network is “I have a hard time controlling my temper”. These differences may stem from developmental and sociocultural factors. From a developmental perspective, adolescent males exhibit higher testosterone levels linked to dominance-seeking and impulsive behaviors, which may amplify physical aggression(Archer 2006). In contrast, females’ earlier pubertal timing and heightened emotional sensitivity could predispose them to internalize distress as anger or hostility(Brix et al. 2019; Holder and Blaustein 2014; Yonkers and Simoni 2018). Culturally, rural Chinese families often reinforce traditional gender norms: males are expected to enter the workforce while females assume primary responsibility for domestic duties(Zhang and Liu 2022). Despite these differences, the core symptoms in the network remain hostility and anger in aggressive behavior. In response to this situation, raising awareness of adolescent mental health, providing early psychological counseling and treatment for those with more serious cases of hostility and anger, and helping students develop positive and healthy values and worldviews during adolescence can alleviate the adverse effects of this symptom(Gilgoff et al. 2020).

These findings will also have a greater impact in different ways in reducing the occurrence of aggressive behavior. For example, engaging families in therapy can help repair relational patterns and improve emotional communication, thereby reducing abusive behaviors. Additionally, schools should prioritize accessible counseling services tailored to gender-specific needs. Interventions should focus on improving family dynamics and providing culturally sensitive mental health support to disrupt the trauma-aggression cycle.

Despite these potential practical implications, it is crucial to recognize the study’s limitations. First, inferring causal relationships and dynamic changes between anxiety and depressive symptoms over time was impossible due to the cross-sectional study design used to collect the data. Second, data were collected using self-report questionnaires, which are known to be prone to bias. The items that assess childhood emotional abuse are open to idiosyncratic interpretation and subjectivity. Third, the rural populations selected for this study are all located in one city. The city is at the top economic level compared to the surrounding areas, and using population data from that area alone does not adequately represent the population as a whole. The study will be more practical if data on students from rural regions with varying economic statuses can be collected. Child emotional abuse occurs in all countries and cultures and the future studies should test the associations found here with different, more representative samples.

## Conclusion

Our study provides important insights into the complex association between emotional abuse and aggressive behavior in adolescents. This network analysis revealed that “Sometimes I feel like people are laughing at me behind my back” and “I have a hard time controlling my temper” as the most central symptoms among network model. As a result, these may be crucial symptoms in developing or maintaining co-occurring emotional abuse and aggressive behaviors in this population. Additionally, multilevel interventions aimed at bridging symptoms such as “Someone in my family has said insulting or sad things to me” and “My parents wish they had never had me” may help to alleviate emotional abuse and aggressive behaviors in this population. This study concluded that psychological sensitivity, negative emotions such as low self-esteem, and poor family relationships that result from adolescents being in rural areas could lead to increased trauma and aggression.

## Electronic supplementary material

Below is the link to the electronic supplementary material.

Supplementary Material 1

Supplementary Material 2

Supplementary Material 3

Supplementary Material 4

## Data Availability

The datasets used and/or analysed during the current study are available from the corresponding author on reasonable request.

## Abbreviations

CTQ-SF: Childhood trauma questionnaire-short form
GGM: Graphical Gaussian model
LASSO: Least absolute shrinkage and selection operator
EBIC: Extended Bayesian information criterion
CS-C: Correlation stability coefficient
CI: Confidence interval
NCT: Network comparison tests
SD: Standard deviation
