# Supplementary material for: The interrelationship between childhood emotional abuse and aggressive behavior in the Chinese adolescent population: a network perspective
Source: Child Adolesc Psychiatry Ment Health. 2025 Jun 14;19:68. doi: 10.1186/s13034-025-00931-3 (PMC12166585; doi:10.1186/s13034-025-00931-3)
Supplement: Supplementary file 1 — Supplementary Material 1 [file 13034_2025_931_MOESM1_ESM.docx]

**Supplementary Material**

**Supplementary figure and table legends**

**Supplementary Table 1: The symptom represented by the node abbreviation.**

**Supplementary Figure 1: Bootstrapped confidence intervals of edge weights.**

**Supplementary Figure 2: Non-parametric bootstrapped difference test for strength.**

**Supplementary Figure 3: Estimation of edge weight difference by bootstrapped difference test.**

**Supplementary Figure 4: Network modeling for males and females.**

**Supplementary Figure 5:** **Comparison of differences in networks by gender**

**The** **correlation coefficients of each node in the network analysis are shown in the Supplementary** **file.**

**Supplementary file 1: Correlation coefficients of each node in the network analysis of full participants.**

**Supplementary file 2: Correlation coefficients of each node in the network analysis of male participants.**

**Supplementary file 3: Correlation coefficients of each node in the network analysis of female participants.**

**Supplementary Table 1: The** **symptom represented by the node abbreviation.**

| Nodes | **Symptom** |
| --- | --- |
| E1 | Some people in the family are called "pig", "ugly" and other unpleasant terms |
| E2 | My parents wish they had never had me |
| E3 | Someone in my family has said insulting or sad things to me |
| E4 | Someone in my family hates me |
| E5 | My mind was tortured or mistreated |
| P1 | If someone provokes me, I might hit him |
| P2 | I've threatened people I know |
| P3 | If someone pushes me hard, I hit him or her |
| P4 | I have been so angry that I have beaten and broken things |
| P5 | I sometimes hit people uncontrollably |
| P6 | I fight more than most people |
| P7 | If someone hits me, I'm going to hit back |
| P8 | If necessary, I will defend my rights by force |
| V1 | My friends say I'm argumentative |
| V2 | I often feel that I disagree with others |
| V3 | When others disagree with me, I can't help but argue with others |
| V4 | I warn people when they interfere with me |
| V5 | When I disagree with my friends, I will be very open and say so |
| I1 | If I am angry, I will deliberately disrupt other people's work |
| I2 | When I'm angry and leave a room with someone, I slam the door |
| I3 | When people dictate to me to do what they want, I muddle through |
| I4 | Sometimes I spread rumors about people I don't like |
| I5 | When someone really annoys me, I ignore him or her |
| I6 | I like playing pranks |
| A1 | I'm prone to sudden anger, but I calm down quickly |
| A2 | Sometimes I get angry for no reason |
| A3 | I have a hard time controlling my temper |
| A4 | I'm a quiet person |
| A5 | I get angry when I don't get what I want |
| A6 | A lot of times I feel like a bomb going off |
| A7 | Some of my friends think I'm a hothead |
| H1 | I think other people always seem to have good chances, good luck |
| H2 | Sometimes, I think life is unfair to me |
| H3 | I don't know why I feel so miserable sometimes |
| H4 | When people are nice to me, I wonder if they want something from me |
| H5 | Sometimes I feel like people are laughing at me behind my back |
| H6 | I don't trust overenthusiastic strangers |
| H7 | I know there are so-called "friends" talking about me behind my back |
| H8 | Sometimes, I can't think about anything else because I'm jealous |

**
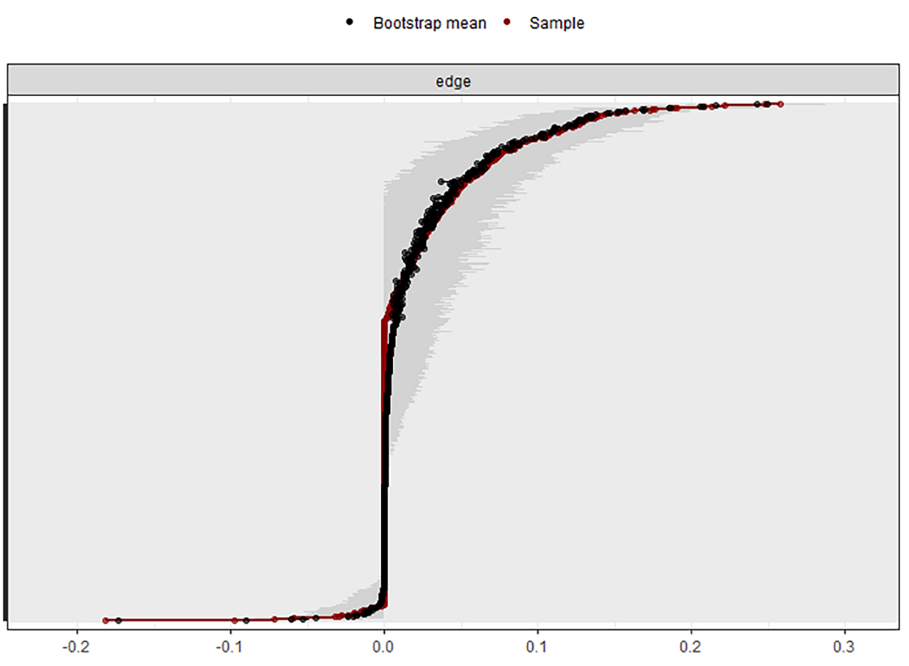
**

**Supplementary Figure 1: Bootstrapped confidence intervals of edge weights.**

The black dots indicate the values of each edge weight, ordered from the highest to the lowest value. The gray area represents the 95% Confidence Intervals of edge weights, estimated with the non-parametric bootstrap procedure (bootnet package). Wide intervals indicate lower stability and narrow intervals indicate higher stability. Because the image vertical coordinates are too dense, only the first and last two coordinates are marked in this paper.


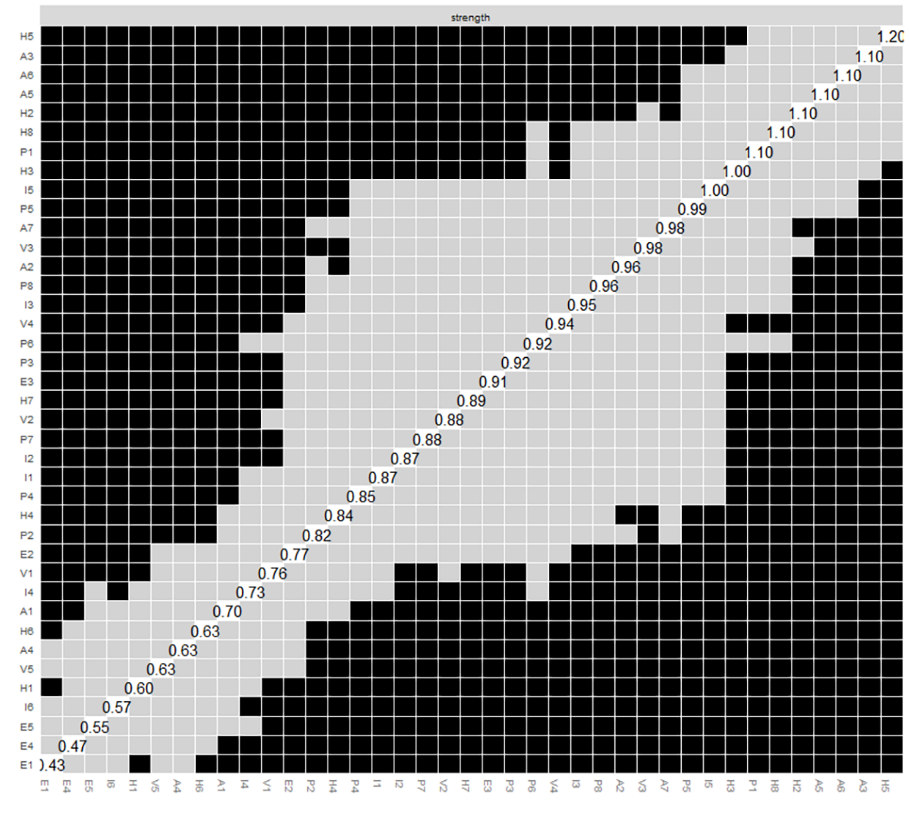


**Supplementary Figure2. Non-parametric bootstrapped difference test for strength.**

Note: Gray boxes indicate no difference between nodes, whereas black boxes indicate a significant difference (α = 0.05). Values reported in the diagonal represent the strength values of each node.


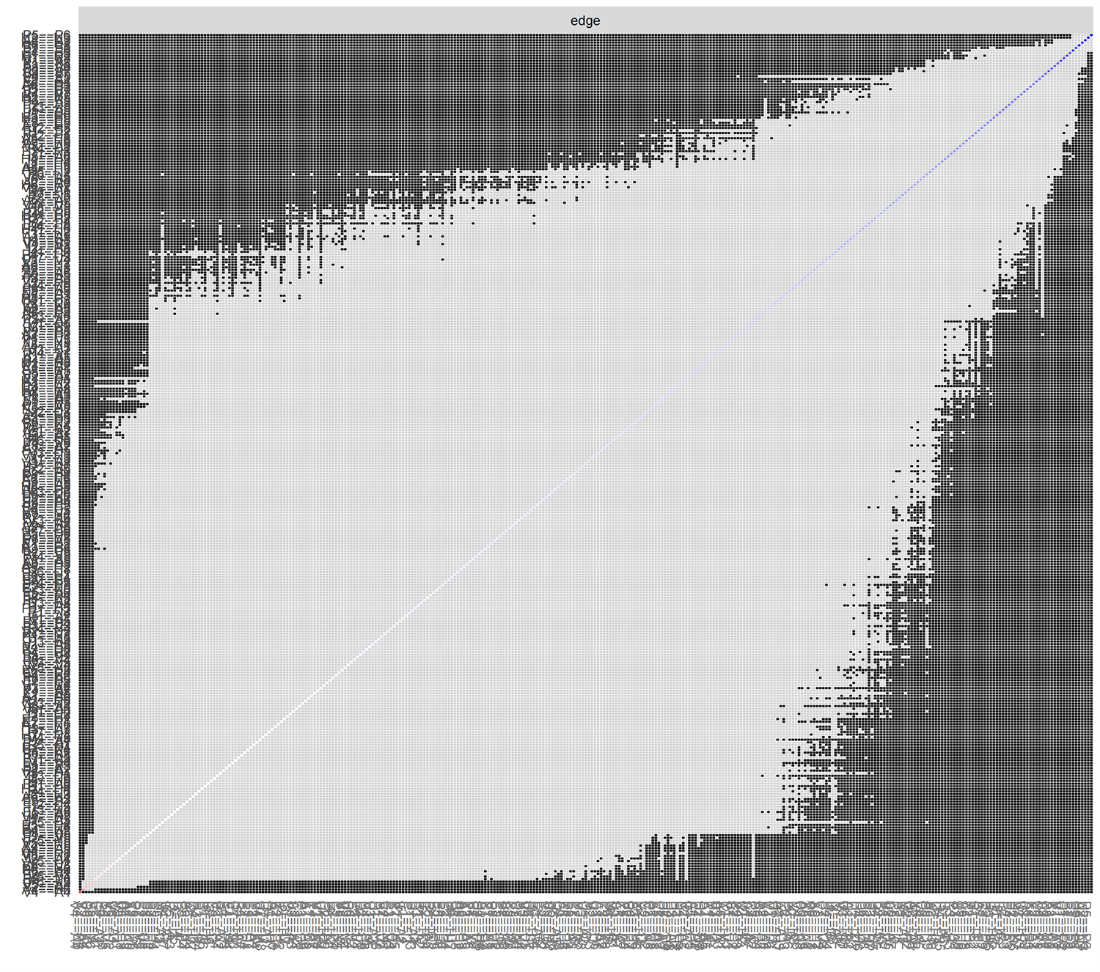


**Supplementary Figure 3: Estimation of edge weight difference by bootstrapped difference test.**

Bootstrapped difference tests between edge weights in the network. Gray boxes indicate edges that do not significantly differ from one-another. Black boxes represent edges with significant difference from one another (α = 0.05). Blue boxes in the edge-weight plot indicate positive correlations.


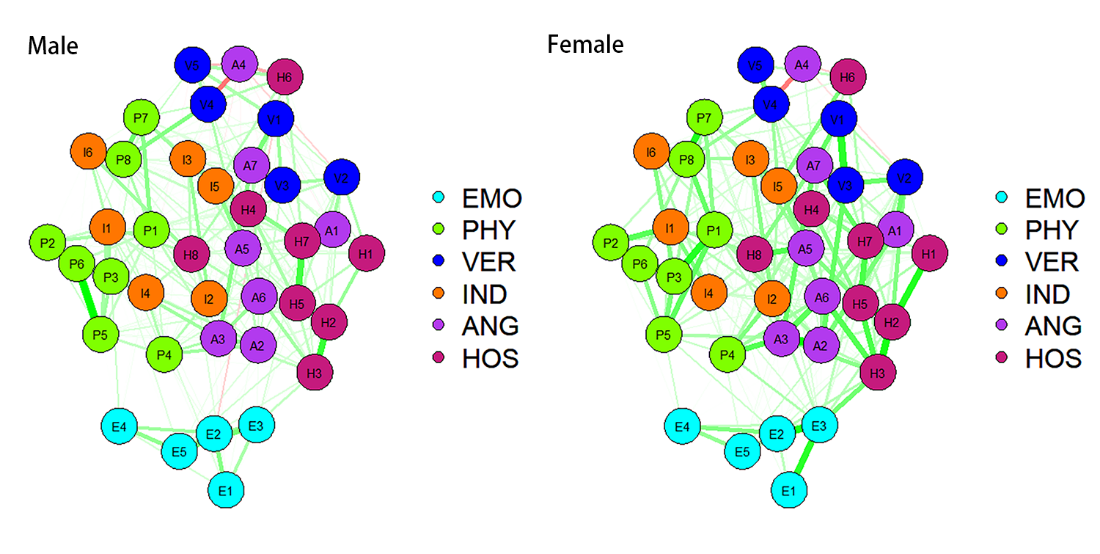


**Supplementary Figure 4: Network modeling for male and female.**


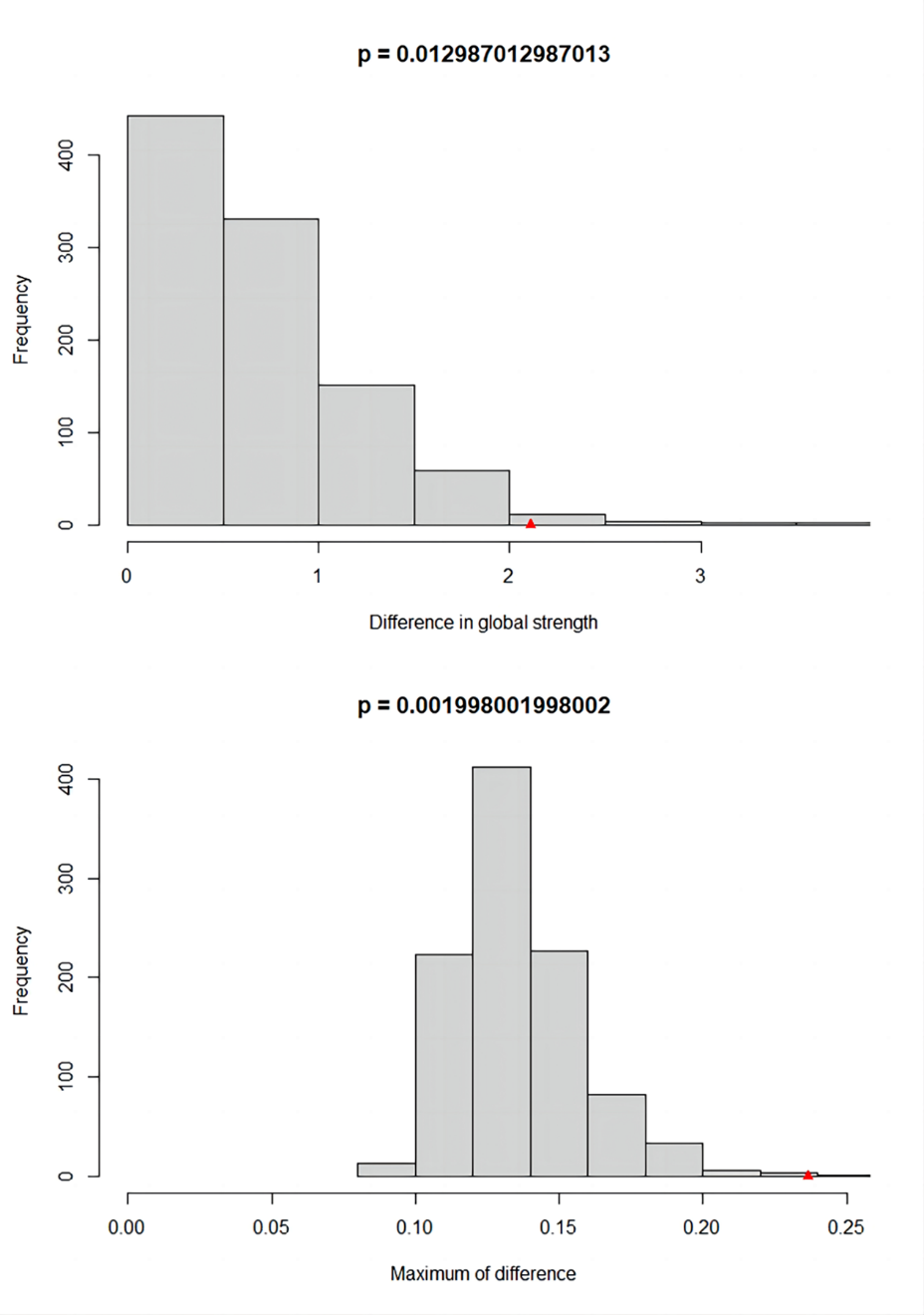


**Supplementary Figure 5:** **Comparison of differences in networks by gender**

There were significant differences in the overall strength of the network(*P*=0.013) and the distribution of edge weights(*P*=0.002) in the comparison of network models.
